# Stressful Life Events and Psychosomatic Symptoms in Fibromyalgia Syndrome and Rheumatoid Arthritis

**DOI:** 10.3390/ijerph22030366

**Published:** 2025-03-02

**Authors:** Ada Ghiggia, Valentina Tesio, Fabrizio Colonna, Enrico Fusaro, Giuliano Carlo Geminiani, Lorys Castelli

**Affiliations:** 1 Department of Life Sciences, University of Trieste, 34128 Trieste, Italy; 2 Department of Psychology, University of Turin, 10124 Turin, Italy; fabriziocolonna14@gmail.com (F.C.); giulianocarlo.geminiani@unito.it (G.C.G.); lorys.castelli@unito.it (L.C.); 3 Rheumatology Unit, “Città Della Salute e Della Scienza” Hospital of Turin, 10126 Turin, Italy; efusaro@cittadellasalute.to.it; 4 Clinical Psychology Unit, “Città Della Salute e Della Scienza” Hospital of Turin, 10126 Turin, Italy

**Keywords:** chronic pain, fibromyalgia, rheumatoid arthritis, psychosomatic syndromes, traumatic life events

## Abstract

Objective: The study analyzed the role of traumatic experiences and psychosomatic components as potential predictors of the likelihood of chronic pain patients having or not having fibromyalgia. Methods: We examined the role of stressful life events (Traumatic Experiences Checklist), psychosomatic syndromes (Toronto Alexithymia Scale and Diagnostic Criteria for Psychosomatic Research), pain, and psychological distress (Beck Depression Inventory—II and State-Trait Anxiety Inventory) in 104 patients with fibromyalgia compared with a sample of 104 patients with rheumatoid arthritis. Results: Patients with fibromyalgia reported significantly more traumatic events, a higher prevalence of psychosomatic syndromes, and higher levels of pain, anxiety and depressive symptoms compared with patients with rheumatoid arthritis (all *p* < 0.01). Hierarchical binary logistic regression with group membership as the dependent variable showed that somatization syndromes (OR = 3.67), pain (OR = 1.56), and childhood trauma (OR = 1.11) were statistically significant predictors of group belonging, and the model explained 67% of the variance in diagnosis [χ^2^(9) = 143.66, *p* < 0.001]. Conclusion: These results highlighted that patients with fibromyalgia are characterized primarily by marked somatization and a high prevalence of early stressful life events compared with patients with rheumatoid arthritis, a primarily nociceptive chronic pain condition. A better knowledge of these mechanisms could allow clinicians to develop tailored interventions that take greater account of the psychological dimension of the disease.

## 1. Introduction

Fibromyalgia (FM) is defined as a complex syndrome characterized by chronic, widespread, non-inflammatory musculoskeletal pain [1]. Chronic pain in FM is often associated with a heterogeneous group of other symptoms, such as sleep disturbances, fatigue, cognitive dysfunction, affective disorders, hyperalgesia, and allodynia, which severely impair the ability to perform daily activities and reduce the patient quality of life [2]. Fibromyalgia is considered the third most common musculoskeletal disorder after low back pain and osteoarthritis, with an estimated worldwide prevalence of 2–3%, affecting women (3.98%) more often than men (0.01%) [3,4].

Although several factors, from genes to stress exposure, have been implicated as predisposing factors, the etiology and pathophysiology of FM are still unknown [3,4,5]. One of the best supported pathophysiological hypotheses is based on central pain sensitization and deficits in endogenous pain inhibitory mechanisms, while some evidence also supports the presence of a peripheral neuropathy affecting small and large fibers [3,6,7]. The lack of objective markers complicates the diagnostic process. It relies mainly on clinical assessment and patients’ reports of subjective symptoms, hindering the understanding and social acceptance of the disease and leading affected patients to experience stigmatization, isolation, and lack of validity of their diagnosis [3,8,9]. To give validity to pain complaints without clear evidence of nociceptive or neuropathic involvement, the term nociplastic pain has recently been proposed [10]. Nociplastic pain can be defined mechanistically as pain resulting from altered function of pain-related sensory pathways in the periphery and central nervous system, causing increased sensitivity [10]. Nociplastic pain can occur in isolation (as in FM) or as a comorbidity in individuals with primary nociceptive or neuropathic chronic pain conditions (e.g., autoimmune diseases such as osteoarthritis) [10,11,12].

Patients with nociplastic pain often report a history of psychosocial trauma and stressful life events [13,14,15]. Early childhood stress occurring during vulnerable periods of life may interact with genetic factors and influence epigenetic mechanisms, leading to structural and functional neuropsychobiological changes and a permanently impaired responsiveness of the allostatic system to repeated or chronic stressors, which could mediate the risk of chronic disease and increase physical and psychological morbidity in adulthood [16,17].

Several studies have reported an association between emotional trauma and FM, although significant differences in the prevalence of past stressful life events have been reported, possibly due to methodological variability [18].

Moreover, patients with FM often report a high prevalence of comorbid psychiatric symptoms, both at clinical and subclinical levels [19,20]. Indeed, growing awareness has demonstrated that subclinical psychopathology and psychosocial stress variables contribute to the global burden of disease, affect quality of life, and have pathophysiological and therapeutic implications that impact treatment outcomes [21]. This awareness has led to the development of valued operational tools for the assessment of those psychosocial variables derived from psychosomatic research that have been shown to be associated with medical disorders, such as the Diagnostic Criteria for Psychosomatic Research (DCPR) [21,22].

To further elucidate the role of traumatic life events and psychosomatic syndromes in chronic nociplastic pain, we investigated the prevalence and potential group predictive ability of stressful life events and psychosomatic syndromes in a group of patients with FM compared to sample of patients with rheumatoid arthritis (RA), a primary nociceptive chronic pain disorder. RA is a chronic, systemic, inflammatory autoimmune disease that affects the synovial membrane of multiple symmetrical joints and leads to chronic pain [23]. The causes are not yet fully understood, but complex interactions between genetic susceptibility, immunological and inflammatory processes, and environmental factors contribute to the risk and progression of RA.

Numerous studies on the psychosocial aspects of FM have compared FM patients with those diagnosed with RA. This comparison is common, as both conditions are a major cause of musculoskeletal pain and disability and have several associated consequences, including pain, fatigue, sleep disturbances, and significant difficulties with daily activities. However, a key difference is that RA has a well-defined pathophysiology and measurable clinical markers for diagnosis, whereas FM has no such clear biological indicators [24]. On this basis, the present study aims to analyze the role of traumatic experiences and psychosomatic components as potential predictors of the likelihood that patients with chronic pain have fibromyalgia, a primarily nociplastic pain disorder, or rheumatoid arthritis, a primarily nociceptive chronic pain disorder.

## 2. Materials and Methods

All FM patients were consecutively recruited in the Clinical Psychology Unit of the Hospital “Città della Salute e della Scienza” in Turin. One hundred and ten female patients gave their consent to participate in the study. Fibromyalgia was diagnosed by an experienced rheumatologist according to the criteria of the American College of Rheumatology [1]. Six women were excluded from the analysis due to a large amount of missing data.

Patients with RA were recruited during routine follow-up at the Rheumatology Unit of the same hospital in Turin. As the FM sample consisted exclusively of women, only female patients with RA without a comorbid FM diagnosis were included in the study. Of the 124 consecutively screened patients with RA, 104 patients met the inclusion criteria and gave written informed consent to participate in the study.

Exclusion criteria for both groups were (1) neurologic disease; (2) severe psychiatric illness or a current major psychiatric diagnosis (excluded by a psychiatrist); and (3) current trauma-related treatment or a recent (less than 6 months ago) traumatic event. The hospital “A.O.U. Città della Salute e della Scienza” in Turin, Italy, granted Institutional Review Board approval.

### 2.1. Clinical and Psychological Distress Assessment

The Italian version of the Fibromyalgia Impact Questionnaire—Revised Form (FIQ-R) is the most widely used questionnaire for assessing FM patients and evaluating all problems associated with the syndrome [25]. The total score is the sum of three domains (overall function, overall impact, and symptoms), with a maximum score of 100 and a higher score indicating greater disease impact. The FIQ-R questionnaire was adapted to the RA group by replacing the word “fibromyalgia” with “rheumatoid arthritis”, as had been performed in a previous study by Näring [26]. The pain item (included in the symptom scale) was used to measure the degree of perceived pain intensity in the last week (range 0–10, the higher the score, the greater the perceived pain).

The Italian version of the Beck Depression Inventory (BDI-II) is one of the most commonly used self-report scales to assess the severity of depressive symptoms [27]. The total score ranges from 0 to 63: higher scores reflect a higher severity of symptoms.

The Italian version of the State-Trait Anxiety Inventory (STAI, Form Y) is divided into two sections, each consisting of twenty-four Likert-type items. STAI-Y1 measures state anxiety, while STAI-Y2 measures trait anxiety [28]. Each scale has a total score between 20 and 80, with a higher score indicating greater anxiety.

### 2.2. Trauma Assessment

The Traumatic Experiences Checklist (TEC) is a self-report questionnaire about 25 types of potential trauma [29]. Traumatic life events experienced in childhood up to the age of 18 are categorized into different domains (emotional neglect, emotional abuse, physical abuse, sexual harassment, and sexual abuse), and a composite score can be calculated for each domain. Composite scores are calculated using the following information: presence or absence, duration of the traumatic experience (>1 year or <1 year), relationship to the perpetrator(s), perceived severity of the experience, and age of the person at the beginning and end of the traumatic experience. The sum of the composite scores of the five domains (0–69) yielded a Total Composite Score indicating the severity of the maltreatment. In addition, the trauma total score was calculated based on the sum of events experienced by each respondent over their lifetime.

### 2.3. Psychosomatic Assessment

The Toronto Alexithymia Scale (TAS-20) was used to measure alexithymia. The Italian version includes a total score and three subscales [30] reflecting the three facets of the alexithymia construct: Difficulty Recognizing Feelings (DIF), Difficulty Describing Feelings (DDF), and Externally Oriented Thinking (EOT). A higher TAS-20 score indicates a higher level of alexithymia (range 20–100).

The Diagnostic Criteria for Psychosomatic Research (DCPR) is a structured Italian interview consisting of a set of 12 psychosomatic syndromes that can be categorized into the following domains: (1) abnormal illness behaviors (illness phobia, thanathopobia, health anxiety, and illness denial); (2) somatization syndromes (persistent somatization, functional somatic symptoms secondary to a psychiatric disorder, conversion symptoms, and anniversary reactions); (3) irritability (irritable mood and type A behavior); (4) alexithymia; and (5) demoralization [31].

### 2.4. Statistical Analysis

Data were analyzed using the Statistical Package for Social Science (IBM Corp. Released 2021. IBM SPSS Statistics for Macintosh, Version 28.0. Armonk, NY, USA: IBM Corp). Student’s *t*-tests for two independent samples and χ^2^ tests were performed to determine whether there were differences between groups on demographic, clinical, and psychological variables. Cohen’s d, r, or φ were calculated to assess effect sizes. For non-normally distributed variables, the Mann–Whitney U test for two independent samples was used. A p-value of less than 0.05 was considered statistically significant.

A hierarchical binary logistic regression analysis was performed, with the FM and RA groups as dependent variables. In accordance with the results of the previous analyses, only those factors (independent variables) for which group membership differed were included in the regression model. Pain, anxiety, and depression symptoms, trauma total score (TEC), composite total score (TEC), and psychosomatic syndromes (DCPR abnormal illness, somatization, irritability, and demoralization) were used as continuous independent variables.

The linearity of the continuous independent variables with respect to the logit of the dependent variable was assessed using the Box–Tidwell procedure [32]. Multicollinearity was controlled using the variance inflation factor (VIF); all values were below 3. Outliers were identified using Cook’s distance: there were no significant differences between the sample with outliers (data are reported in the following sections) and without outliers. Adjusted odds ratios (ORs) and 95% confidence intervals (CIs) of the predictors were measured.

## 3. Results

### 3.1. Socio-Demographic and Clinical Variables

The socio-demographic and clinical data are shown in Table 1. No significant differences were found between FM and RA in terms of age or years of education.

The majority of patients in both groups were married (56.3% of FM and 71.2% of RA) and currently employed (62.5% of FM and 51.9% of RA). The clinical data (FIQ-R and pain intensity) showed a worse impact of the disease on the quality of life of FM patients (FIQ-R) compared to RA patients (all *p* < 0.001).

Looking at the results of the BDI-II and the STAI-Y, patients with FM reported more depressive and anxiety symptoms than patients with RA, as shown by the independent samples *t*-tests (*p* < 0.001) in Table 1.

### 3.2. Trauma Assessment

The data on traumatic life experiences are presented in Table 2. Given the non-normal distribution of the data, the Mann–Whitney U test was used. FM patients reported more traumatic life experiences, with statistically significant differences in the Total Composite Score and in each subscale of the TEC assessing the different types of traumatic events from 0 to 18 years of age (*p* < 0.05), as well as in the trauma total score (*p* < 0.001).

Regarding the total trauma score, which assesses the presence of trauma across the lifespan, 99% of patients in both groups reported at least one traumatic event in their lifetime. Specifically, 54% of FM patients versus 19% of RA patients reported experiences of emotional neglect by family members (parents and/or siblings) in their lives; 43% of FM and 11% of RA patients also reported experiences of emotional abuse by their family members. About half of FM patients (41%) experienced role reversal (having to care for their parents and/or siblings as a child), compared to 28% of RA patients.

### 3.3. Psychosomatic Assessment

Regarding alexithymia, the comparison of the three factors of the TAS-20 questionnaire between the two groups (independent samples *t*-test) showed that the factor difficulty in identifying feelings was statistically significantly higher in the FM patients than in the RA group (*p* < 0.001), although no statistically significant difference was found in the TAS-20 total score (Table 1). In the FM sample, 35.6% of patients are above the TAS-20 cutoff and 20.3% are in the subclinical range. In the RA group, 22.1% are above the threshold for alexithymia and 26% are in the subclinical range.

The results of the DCPR are shown in Table 2. About one-fifth of the RA sample (18%) did not meet the criteria for the psychosomatic syndromes, whereas every FM patient had at least one psychosomatic syndrome. FM patients primarily reported persistent somatization (79% in FM vs. 20.9% in RA), type A behavior (58.7% in FM vs. 36.5% in RA), and demoralization (51% in FM vs. 18.3% in RA). No statistically significant differences (*p*-value > 0.05) were found for health anxiety, disease phobia, illness denial, and alexithymia.

### 3.4. Regression Analysis

A binomial logistic regression was performed to determine the effects of pain, psychological distress, psychosomatic syndromes, and trauma on the likelihood of patients having FM or RA (Table 3).

Of the nine predictor variables, only three were statistically significant: pain (β = 0.441, *p* < 0.001), Total Composite Score (β = 0.100, *p* = 0.012), and DCPR somatization (β = 1.298, *p* < 0.001) (Table 3). As the pain level increased, the likelihood of being classified in the FM group was 1.56 times higher (95% CI = 1.26–1.92). Increasing values of the composite trauma score were associated with a higher likelihood of having a diagnosis of fibromyalgia (OR = 1.01; 95% CI = 1.02–1.20). Similarly, those who reported more DCPR somatization syndromes were 3.67 times more likely to be classified in the FM group (95% CI = 2.24–5.98). The model was statistically significant [χ^2^(9) = 143.66, *p* < 0.001] and explained 67% (Nagelkerke R^2^) of the variance in diagnosis, and 82.6% of cases were correctly classified.

## 4. Discussion

The aim of the present study was to delve deeper into the role of traumatic life experiences and psychosomatic syndromes in a sample of FM patients by comparing them with a matched group of women with RA, a primary nociceptive chronic pain disorder. Specifically, we investigated which of these factors could be considered the best potential predictors of the likelihood that a person would or would not have fibromyalgia.

Although previous studies have examined the prevalence of stressful life events in fibromyalgia [5,26], there are fewer studies that have also examined somatization and psychosocial stress variables in the same disease [33] or more generally in chronic pain disorders [17]. As far as we are aware, no study has simultaneously considered traumatic life events and psychosomatic syndromes in fibromyalgia. In addition, the comparison with a matched group of rheumatoid patients with RA allowed us to compare the differences between two types of chronic pain disorders: a primary nociplastic and a primary nociceptive pain disorder.

In terms of clinical assessment, FM patients reported worse symptomatology and physical functioning, as well as a greater impairment of their quality of life due to the disease compared to the RA group. These findings are consistent with previous studies highlighting the more severe impairments caused by FM in all aspects of daily life and often associated with lower work productivity [23]. Regarding pain, patients with FM had a higher pain intensity, which is easily explained by the nociplastic pain characteristic of this syndrome [10]. Recent empirical studies have shown that patients with FM show greater salience and attention to pain when exposed to painful pressure stimuli compared to patients with RA [34]. Nociplastic pain tends to fluctuate in terms of location and intensity and is often exacerbated not only by physical activity or environmental stimuli (e.g., climate change) but also by psychological distress.

The close association between chronic pain and psychological distress is well established in the literature, with a high prevalence of depression and anxiety in people with chronic pain [35,36]. Levels of anxiety and depressive symptoms in patients with RA are higher than in the general population [37], with a prevalence of 20–70% for depressive symptoms [38] and around 45% for anxiety [39]. Despite these high comorbidities in RA, and in line with previous studies [40], our results confirmed that FM patients had higher levels of clinically relevant depressive and anxiety symptoms compared to patients with RA [41].

As noted in a recent systematic review of precipitating traumatic events in FM [18], although a large-scale meta-analysis found a clear association between psychological trauma and FM [5], the overall low quality of evidence due to methodological weaknesses, the most important of which is recall bias, is a confounding factor. Indeed, chronic pain and psychological disorders have been shown to increase recall of negative life events [18]. The use of RA as a control group for chronic pain provides further evidence that the association found between psychological trauma and FM is not simply due to recall bias. In fact, the data on traumatic experiences showed that although almost everyone has experienced at least one or more traumatic situations in their lives, regardless of disease, patients with FM experienced a significantly higher number of negative life events compared to RA.

Although the majority of studies agree that traumatic life events may be a predictor of the onset and development of FM, the debate about the nature and timing of trauma remains controversial and leads to conflicting results [5,18]. Looking more closely at the typology of traumatic childhood events experienced by the patients in our sample, we found that emotional neglect and emotional abuse were very common in early childhood (0–18 years). In fact, we found that these adverse events were significantly more common than in the RA sample, a finding that is consistent with a previous research study that used similar control groups [26].

Traumatic experiences may not only contribute to the development and exacerbation of chronic pain and psychological distress [16,42] but may also lead to the development of features of emotional dysregulation (e.g., alexithymia) [43]. Indeed, the literature on the presence of alexithymia in chronic pain patients is extensive [44], and alexithymia is known to impair adherence and compliance in many cases. Our TAS-20 results showed comparable and high levels of alexithymia in both samples, with more than one-third of patients with FM and one-fifth of patients with RA scoring above the cut-off point for alexithymia. These results were confirmed by the DCPR data, which showed only a slightly higher prevalence of alexithymia in RA compared to TAS-20 (26% and 22%, respectively). These findings are consistent with recent evidence showing that patients with chronic pain, whether nociceptive, neuropathic, or nociplastic pain, have greater difficulties with emotion regulation than non-clinical and clinical samples without pain [45,46].

While alexithymia has been widely studied in chronic pain conditions, years of psychosomatic research have shown that there are several other psychosocial stress variables that contribute to the global burden of somatic symptoms and medical illness and have corresponding pathophysiologic and therapeutic implications [21]. Although several articles focused on single dimensions such as somatization [47] or health anxiety [42], a simultaneous assessment of all these aspects and a comparison with patients with chronic pain conditions of other origins would provide a more comprehensive overview. In our sample, every patient with FM reported at least one of the twelve psychosomatic syndromes assessed with the DCPR. In contrast, about one-fifth of the patients in the RA group did not fulfill the criteria for any of the syndromes. Although the groups differed in terms of abnormal illness behaviors, only thanatophobia was found to be significantly more common in patients with FM compared to the RA group. Irritability (type A behavior and irritable mood) was reported to a high degree in both samples (about 30% of RA and 50% of FM patients), but with a significantly higher prevalence in FM than in AR patients. Demoralization was also highly prevalent in the FM sample (more than half of the patients with FM). Recent research has shown that demoralization plays a role not only in cancer patients [48], but also in chronically ill patients [49]. Indeed, demoralized individuals with chronic diseases may experience stress and subjective incompetence because they are “shackled” to a disease and its treatment, which, if not properly recognized and treated, can lead to feelings of hopelessness and helplessness [49]. These feelings can be even more pronounced in FM patients who also struggle with the lack of validity and social acceptance of chronic nociplastic pain symptoms and diagnoses.

Finally, as expected, the patients with FM reported a high prevalence of psychosocial stress variables in the somatization syndromes domains, with approximately 80% affected by persistent somatization, 50% by conversion symptoms, and 45% by anniversary reactions, all percentages significantly higher than those of the patients with RA.

Somatization was defined as “the tendency to experience and communicate somatic complaints and symptoms that are not justified by pathological findings, to attribute them to a physical illness, and to seek medical help for them” [50]. In some cases, it could be argued that somatic symptoms in chronic pain conditions (such as pain in FM) are an expression of emotional distress that patients deny by focusing on the purely somatic symptom and neglecting the emotional component. In addition, personal characteristics and the experience of traumatic events throughout life, exacerbated by the possible resulting dysregulation of emotions (e.g., alexithymia), may contribute to the development and maintenance of somatization.

To further explore the role of traumatic events and psychosomatic syndromes, in addition to assessing and comparing their prevalence in the two chronic pain conditions, we investigated which of the factors that differ significantly between the two conditions best contribute to group membership/disease. We found that, in addition to pain, only somatization syndromes and childhood trauma (Total Composite Score of the TEC) were significant predictors of a person receiving a diagnosis of fibromyalgia rather than rheumatoid arthritis. Taken together, neither the differences in psychological distress nor the differences in the other types of psychosomatic syndromes contribute significantly to explaining the differences between the groups. The non-significant predictive role of anxiety and depression symptoms indicates their clinical importance in both pathologies and confirms the close relationship between chronic pain and psychological distress, regardless of the causes and nature of the pain itself [51]. From a clinical perspective, the fact that psychological factors other than psychological distress, such as somatization, play a more central role in FM underlines the importance of assessing these aspects in order to implement appropriate and effective psychological interventions [52].

Overall, our results confirmed that FM patients are primarily characterized by marked somatization and a high prevalence of early stressful life events compared with a similar chronic condition in which pain is primarily nociceptive in origin, such as RA.

Traumatic experiences in childhood can have profound and lasting effects on coping skills, illness behavior and symptom perception [53]. In addition, they may lead to changes in the neural and neurohormonal systems involved in pain modulation, possibly through a process of central sensitization [53], which is one of the best-documented factors involved in the pathophysiology of FM. Chronic nociplastic pain is, indeed, characterized by a central sensitization that increases the perception of pain even in response to harmless stimuli. This hypersensitivity is modulated by both peripheral factors, such as inflammation, and central factors, including cognitive and emotional processes [24]. Stress, whether physical or psychological, has further effects on these systems by affecting the immune response, the sympathetic nervous system, and the hypothalamic–pituitary–adrenal axis. Traumatic experiences in childhood have also been associated with somatization [54]. Although the exact mechanisms linking early trauma to somatization remain unclear, several hypotheses have been proposed that bodily symptoms may serve as an expression of emotional suffering [54]. In terms of neurophysiological aspects, there are common pathways involving altered body awareness and central nervous system dysregulation, which can increase pain and vulnerability to illness [55].

Although the long-standing association between childhood trauma and somatization is widely recognized, future research should further investigate the underlying neurobiological mechanisms in fibromyalgia, which remain controversial and unknown [50]. In particular, it appears that in nociplastic pain disorders, stressful life events (particularly in early childhood and possibly in combination with predisposing genetic factors) may influence epigenetic mechanisms leading to structural and functional neuropsychobiological impairment in the responsiveness of the allostatic system to repetitive or chronic stressors, resulting in increased somatization and contributing to exacerbate the chronic pain condition itself [24].

There are some methodological limitations in our study that should be taken into account when interpreting the results. First, due to the cross-sectional design of the study, it is unfortunately not possible to determine causal directions; future longitudinal studies are needed to clarify the temporal relationship between trauma and FM and to determine whether trauma plays a causal role in its development. Second, because FM occurs predominantly in women, we were unable to recruit male patients with FM and the entire sample consisted exclusively of women, so the results cannot be generalized to the male population. Third, the present study did not examine the role of other potentially important variables, such as attachment style, in the relationship between trauma and psychosomatic syndromes. Given the predominant role of emotional neglect and abuse, more detailed information should be considered to better elucidate the role of traumatic history in symptom onset. Finally, a comparison between FM and a psychiatric control group (e.g., patients with depressive disorders) could clarify the psychological components of FM, distinguishing them from those typical of mood disorders.

In conclusion, our results underline the strong presence of psychosomatic syndromes, especially somatization, and traumatic life events in fibromyalgia patients. A better knowledge and understanding of mechanisms underlying somatization and their role in the development and maintenance of fibromyalgia could allow the development of more specific and individualized interventions. Appropriate psychotherapeutic treatment may be essential not only to reduce clinical symptoms but also to reduce the perception of pain by improving the ability to distinguish emotional states from physical sensations.

## Figures and Tables

**Table 1:** Socio-demographic, clinical and psychological variables, mean (±SD), number (percentage), comparisons (independent samples *t*-test) between groups and effect size are reported.

	FM (N = 104)	RA (N = 104)	*t*-Test (df)	*p*	*d*
**Age (years)**					
Range	23–70	22–70	−1.84 (205)	0.067	
Mean ± SD	50.8 (9.8)	53.3 (10.1)			
**Education (years)**					
Mean ± SD	11.8 (3.4)	11.3 (3.7)	1.01 (206)	0.313	
**Marital status**				0.089	
Single	11 (10.6)	11 (10.6)			
Cohabitant	12 (11.7)	6 (5.8)			
Married	58 (56.3)	74 (71.2)			
Divorced	16 (15.5)	12 (11.5)			
Widowed	6 (5.8)	1 (1.0)			
**Work status**				0.196	
Students	2 (1.9)	2 (1.9)			
Employed	65 (62.5)	54 (51.9)			
Housewife	17 (16.3)	18 (17.3)			
Not employed	11 (10.6)	9 (8.7)			
Retired	9 (8.7)	21 (20.2)			
**FIQ-R Total score**	63.4 (17.7)	35.8 (21.6)	10.08 (197.98)	<0.001	−1.40
Physical Functioning	17.2 (6.5)	9.6 (7.3)	7.90 (205)	<0.001	−1.10
Overall Impact	11.9 (5.9)	6.6 (5.5)	6.71 (205)	<0.001	−0.93
Symptoms	34.3 (7.5)	19.5 (10.8)	11.48 (183.74)	<0.001	−1.60
Pain	7.5 (1.8)	4.5 (2.7)	9.57 (182.27)	<0.001	−1.33
**Beck Depression Inventory-II**	19.6 (9.4)	11.8 (8.3)	6.377 (206)	<0.001	−0.88
**STAI—State form Y**	43.3 (12.4)	36.9 (10.7)	3.968 (199.76)	<0.001	−0.55
**STAI—Trait form Y**	51.9 (10.9)	42.9 (9.4)	6.387 (206)	<0.001	−0.89
**Alexithymia (TAS-20)**					
TAS-20 Total score	53.6 (14.0)	50.0 (13.9)	1.845 (206)	0.066	−0.26
TAS—DIF	22.1 (7.3)	17.4 (7.5)	4.629 (206)	<0.001	−0.64
TAS—DDF	14.0 (5.3)	13.6 (4.7)	0.681 (203.09)	0.497	−0.94
TAS—EOT	17.4 (5.2)	19.1 (4.9)	−2.352 (206)	0.020	0.33

Abbreviations: FM: fibromyalgia; RA: rheumatoid arthritis. FIQ-R: Fibromyalgia Impact Questionnaire—Revised Form; STAI: State-Trait Anxiety Inventory Form Y; TAS-20: Toronto Alexithymia Scale 20-items; DIF: difficulty in identifying feelings; DDF: difficulty in describing feelings, EOT: externally oriented thinking.

**Table 2:** Mean (SD), frequencies (%), and comparison between FM (fibromyalgia) and RA (rheumatoid arthritis) patients are reported for the Traumatic Experiences Checklist (TEC) and the Diagnostic Criteria for Psychosomatic Research (DCPR).

		**Mean (±SD)**		**Z-Test**	***p***	**Effect Size**
		**FM** **(N = 104)**	**RA** **(N = 104)**			**r**
**Traumatic Experiences Checklist**						
	Emotional Neglect	4.24 (4.72)	1.26 (2.98)	−5.274	<0.001	0.37
	Emotional Abuse	3.22 (4.15)	0.77 (2.36)	−5.216	<0.001	0.36
	Physical Abuse	1.33 (3.08)	0.36 (1.29)	−2.457	0.014	0.17
	Bodily Threat	0.96 (1.89)	0.34 (0.98)	−2.869	0.004	0.20
	Sexual Harassment	0.72 (1.61)	0.21 (0.66)	−2.888	0.004	0.20
	Sexual Abuse	0.42 (1.59)	0.06 (0.34)	−2.255	0.024	0.16
	Total Composite Score	10.77 (10.75)	2.94 (6.35)	−6.911	<0.001	0.48
	Trauma Total Score	6.22 (3.73)	3.81 (2.81)	−5.083	<0.001	0.35
**Diagnostic Criteria for Psychosomatic Research**		**Frequencies (%)**		**χ² (df)**	***p***	**φ**
		**FM** (N = 104)	**RA** (N = 104)			
**Abnormal illness** **behavior**	Health Anxiety	17 (16.3)	16 (15.4)	0.04 (1)	0.849	0.01
	Disease Phobia	9 (8.7)	3 (2.9)	3.18 (1)	0.074	0.12
	Thanatophobia	13 (12.5)	3 (2.9)	6.77 (1)	0.009	0.18
	Illness Denial	32 (30.8)	22 (21.2)	2.50 (1)	0.114	0.11
**Somatization syndromes**	Functional Symptoms	18 (17.3)	1 (1.0)	16.74 (1)	<0.001	0.28
	Persistent Somatization	68 (79.1)	18 (20.9)	49.56 (1)	<0.001	0.49
	Conversion Symptom	51 (49.0)	8 (7.7)	43.75 (1)	<0.001	0.46
	Anniversary Reaction	48 (46.2)	20 (19.2)	17.13 (1)	<0.001	0.29
**Irritability**	Type A Behavior	61 (58.7)	38 (36.5)	10.20 (1)	0.001	0.22
	Irritable Mood	46 (44.2)	25 (24.0)	9.43 (1)	0.002	0.21
**Demoralization**	Demoralization	53 (51.0)	19 (18.3)	24.56 (1)	<0.001	0.34
**Alexithymia**	Alexithymia	38 (36.5)	28 (26.9)	2.22 (1)	0.136	0.10

**Table 3:** Hierarchical logistic predicting likelihood of fibromyalgia versus rheumatoid arthritis based on pain, anxiety, and depressive symptoms, trauma, and psychosomatic syndromes (N = 208).

Predictor Variables	B	SE	Wald	*df*	*p*	Odds Ratio	95% CI for Odds Ratio	
							Lower	Upper
FIQ-R Pain	0.441	0.108	16.794	1	<0.001	1.56	1.259	1.920
BDI-II	−0.011	0.035	0.095	1	0.758	0.99	0.923	1.060
STAI-Y2	0.055	0.029	3.541	1	0.060	1.06	0.998	1.119
TEC Trauma total score	−0.032	0.095	0.114	1	0.735	0.97	0.804	1.167
TEC Total Composite Score	0.100	0.040	6.250	1	0.012	1.01	1.022	1.195
DCPR Abnormal illness	0.171	0.314	0.296	1	0.587	1.19	0.641	2.196
DCPR Somatization	1.298	0.250	26.909	1	<0.001	3.67	2.243	5.980
DCPR Irritability	−0.298	0.306	0.953	1	0.329	0.74	0.408	1.351
DCPR Demoralization	0.671	0.464	2.088	1	0.148	0.51	0.206	1.270

Abbreviations: FIQ-R: Fibromyalgia Impact Questionnaire—Revised Form. BDI-II: Beck Depression Inventory; STAI: State-Trait Anxiety Inventory Form Y2, Trait questionnaire; TEC: Traumatic Experiences Checklist; DCPR: Diagnostic Criteria for Psychosomatic Research.

## Data Availability

All relevant data are within the manuscript file. The dataset used and/or analyzed during the current study is available from the corresponding author on reasonable request.
